# Supplementary material for: Mapping Local Variations and the Determinants of Childhood Stunting in Nigeria
Source: Int J Environ Res Public Health. 2023 Feb 13;20(4):3250. doi: 10.3390/ijerph20043250 (PMC9959360; doi:10.3390/ijerph20043250)
Supplement: Supplementary file 1 [file ijerph-20-03250-s001.zip › Supplementary Table S1.pdf]

Table S1: Prevalence of childhood stunting across first administrative level of Nigeria, 2022

Administrative states	Prevalence (%)	Lower CrIs (%)	Upper CrIs (%)
Abia	17.10	4.37	50.42
Adamawa	36.79	9.16	76.82
Akwa Ibom	23.92	5.38	62.83
Anambra	20.67	5.19	54.30
Bauchi	51.35	16.19	85.14
Bayelsa	27.51	6.41	67.59
Benue	20.92	4.49	60.16
Borno	55.35	17.65	87.53
Cross River	26.28	5.83	67.11
Delta	26.23	5.99	65.99
Ebonyi	24.25	6.11	61.90
Edo	22.12	4.87	61.03
Ekiti	16.58	3.96	51.09
Enugu	16.97	3.95	51.62
Federal Capital Territory	23.47	5.87	60.08
Gombe	52.78	17.42	85.30
Imo	15.35	3.88	47.80
Jigawa	60.59	21.50	89.53
Kaduna	38.89	10.57	76.90
Kano	52.95	17.27	85.84
Katsina	55.95	18.36	87.66
Kebbi	54.82	17.79	87.20
Kogi	25.47	5.89	65.09
Kwara	42.93	12.10	79.36
Lagos	23.12	5.63	58.99
Nasarawa	29.22	7.03	69.42
Niger	47.90	13.93	83.69
Ogun	25.06	5.72	65.19
Ondo	23.02	5.25	61.50
Osun	17.29	4.11	52.58
Oyo	30.37	7.36	70.35
Plateau	33.63	8.72	72.36
Rivers	22.42	5.04	61.06
Sokoto	61.71	21.65	90.24
Taraba	34.24	8.58	73.81
Yobe	61.99	21.75	90.38
Zamfara	55.46	18.17	87.42

CrIs indicate credible intervals
