# Supplementary material for: Mapping Local Variations and the Determinants of Childhood Stunting in Nigeria
Source: Int J Environ Res Public Health. 2023 Feb 13;20(4):3250. doi: 10.3390/ijerph20043250 (PMC9959360; doi:10.3390/ijerph20043250)
Supplement: Supplementary file 1 [file ijerph-20-03250-s001.zip › Supplementary Table S2.pdf]

Supplementary Table S2: Prevalence of childhood stunting across second administrative level of Nigeria (local government counsils), 2022

Local administration	Prevalence	Lower CrIs	Upper CrIs
Shomolu	2.0	0.5	7.6
Onitsha South	6.5	2.1	18.1
Alimosho	8.2	2.4	26.8
Umuahia South	8.4	2.0	31.6
Nnewi North	8.5	2.9	25.1
Enugu South	8.7	2.8	25.9
Egor	9.2	2.0	32.4
Owerri-Municipal	9.5	1.3	45.5
Agege	9.7	2.1	38.8
Surulere	9.9	5.0	20.5
Ilesha East	10.0	2.1	36.7
Ajeromi-Ifelodun	10.1	5.3	18.9
Oshodi-Isolo	10.5	4.1	30.8
Enugu North	10.5	3.4	32.9
Ikot Ekpene	10.8	2.4	36.4
Njikoka	10.9	4.1	26.9
Idemili South	10.9	3.8	31.5
Oyi	11.0	3.0	30.8
Ilesha West	11.2	2.8	35.0
Mbaitoli	11.3	3.4	34.9
Nnewi South	11.4	2.9	37.8
Ekwusigo	11.7	2.6	39.7
Umuahia North	11.8	2.9	38.2
Obowo	12.0	3.9	35.5
Ojo	12.1	5.1	30.6
Ife Central	12.3	2.6	42.4
Oru East	12.4	3.5	39.1
Ikeduru	12.5	2.4	45.8
Awgu	12.7	2.9	41.7
Boripe	12.7	2.6	45.4
Onitsha North	12.7	3.6	37.7
Uvwie	12.9	3.2	42.1
Aguata	12.9	5.6	34.7
Owerri North	13.0	2.8	43.3
Moba	13.0	3.9	36.2
Isiala Mbano	13.1	2.9	44.4
Obia/Akpor	13.1	3.5	40.2
Idemili North	13.1	5.8	30.8
Anaocha	13.2	3.8	39.4
Ihitte/Uboma	13.3	2.7	46.4
Nkwerre	13.3	5.6	29.7
Ahiazu-Mbaise	13.4	3.5	44.6
Amuwo-Odofin	13.5	4.7	41.7
Port-Harcourt	13.6	4.3	39.9
Isiukwuato	13.7	2.8	46.1
Suleja	13.7	2.8	46.7
Ihiala	13.9	3.1	46.3
Enugu East	13.9	3.1	45.7
Umu-Nneochi	14.0	3.5	45.1
Osogbo	14.1	4.4	40.1
Uyo	14.1	3.1	45.6
Ila	14.1	4.1	41.3
Igbo-Etiti	14.1	3.2	45.8
Ado Ekiti	14.1	3.4	45.8
Esan Central	14.2	2.5	52.0
Jos North	14.2	4.5	41.5
Ehime-Mbano	14.2	3.1	48.6
Ijero	14.3	3.1	47.2
Nkanu West	14.3	3.4	45.0
Unuimo	14.3	4.7	38.4

Ikeja	14.4	4.9	32.2
Ifedayo	14.4	4.4	41.2
Akure South	14.5	3.0	50.7
Orumba North	14.5	3.6	43.3
Obokun	14.5	2.9	48.9
Ekiti East	14.5	2.8	51.7
Olorunda	14.6	3.5	47.0
Obanliku	14.7	2.8	51.8
Oguta	14.7	3.0	51.0
Kosofe	14.8	4.8	40.4
Dunukofia	14.8	3.7	41.6
Okigwe	14.8	3.5	47.9
Oriade	14.8	3.6	47.1
Orlu	14.9	4.2	41.7
Akoko North West	14.9	3.7	45.1
Jos South	14.9	3.5	48.1
Nsukka	15.0	3.2	49.2
Ezinihitte	15.0	4.4	45.1
Ifelodun	15.1	3.7	45.9
Oji-River	15.1	3.2	48.4
Nwangele	15.3	4.4	43.7
Ideato South	15.3	6.1	37.3
Njaba	15.3	5.7	35.1
Abuja Municipal	15.3	3.4	49.5
Orumba South	15.4	3.3	52.0
Esan West	15.4	3.1	51.1
Ilejemeji	15.5	4.4	48.3
Ede North	15.5	3.5	48.9
Ogori/Magongo	15.5	3.5	48.0
Mushin	15.6	10.2	23.9
Igueben	15.6	3.0	52.1
Ife East	15.6	4.2	47.5
Udi	15.6	3.4	50.2
Awka South	15.7	3.8	49.2
Ikere	15.7	4.0	47.7
Isiala-Ngwa South	15.8	4.3	48.0
Oru West	15.8	6.1	39.1
Tarka	15.8	3.6	50.8
Ifedore	15.8	3.2	53.2
Oye	15.8	3.8	47.9
Irepodun/Ifelodun	15.8	4.2	48.7
Apapa	15.8	6.4	35.6
Owerri West	15.9	3.5	50.5
Ilorin West	15.9	5.1	43.4
Ideato North	15.9	5.5	41.9
Ede South	15.9	2.9	55.1
Ibadan South West	16.0	9.4	34.6
Akure North	16.0	3.2	51.6
Ekiti West	16.0	3.8	50.5
Gboko	16.0	3.3	51.5
Ogba/Egbema/Ndoni	16.0	3.3	52.3
Emure	16.0	3.0	52.8
Obot Akara	16.1	3.7	49.6
Orsu	16.1	5.2	41.8
Ikwuano	16.1	3.0	54.6
Akoko South West	16.3	3.8	49.7
Yola North	16.3	3.8	49.2
Udenu	16.4	4.6	48.0
Asari-Toru	16.4	4.5	46.4
Efon	16.5	4.7	51.0
Aboh-Mbaise	16.5	4.9	46.1
Ika North East	16.5	3.2	55.2
Chanchaga	16.6	6.2	38.3
Ido-Osi	16.6	6.7	41.2

Ikorodu	16.6	4.3	50.9
Ohafia	16.6	4.4	46.6
Ohaji/Egbema	16.7	3.7	53.3
Isin	16.7	3.9	51.2
Igbo-Eze South	16.7	5.2	44.4
Ondo East	16.9	4.2	51.5
Ika South	16.9	3.7	52.6
Atakumosa West	16.9	3.7	54.3
Kwande	17.0	3.5	53.8
Aiyekire (Gbonyin)	17.0	4.3	51.6
Abak	17.0	4.3	48.2
Esan North East	17.0	4.1	51.6
Ukwa West	17.0	3.7	53.0
Ife South	17.1	3.8	52.9
Bwari	17.1	4.1	49.3
Ogbadibo	17.2	3.4	56.1
Atakumosa East	17.2	4.2	51.7
Egbedore	17.2	4.8	50.9
Kaura	17.2	3.8	53.4
Isiala-Ngwa North	17.2	5.4	46.5
Ilorin South	17.2	4.5	49.3
Ikenne	17.3	5.2	47.5
Ile-Oluji-Okeigbo	17.3	3.8	53.9
Etsako West	17.4	3.9	52.2
Oluyole	17.4	3.7	52.9
Ekiti South West	17.4	4.3	51.2
Ogbaru	17.4	4.1	51.3
Eti-Osa	17.5	3.0	59.3
Ondo West	17.5	3.5	55.8
Ukwuani	17.6	3.9	53.5
Obi Ngwa	17.6	4.4	51.1
Ikwerre	17.7	4.5	52.0
Irewole	17.7	4.8	49.0
Irepodun	17.8	4.0	53.6
Ohimini	17.9	3.9	54.3
Ikono	17.9	4.2	52.1
Akoko South East	18.0	5.0	48.9
Ise/Orun	18.0	4.5	52.0
Ife North	18.0	3.8	54.5
Aiyedire	18.1	3.7	57.5
Okpokwu	18.1	3.8	56.4
Ahoada East	18.1	4.0	53.8
Ado-Odo/Ota	18.2	4.8	53.1
Igbo-Eze North	18.2	4.6	52.5
Remo North	18.2	4.0	53.3
Ugwunagbo	18.2	4.9	50.1
Eleme	18.2	7.3	40.8
Isi-Uzo	18.3	3.8	56.2
Akoko-Edo	18.3	3.8	55.9
Ijumu	18.3	4.0	55.7
Akinyele	18.3	4.1	54.7
Obudu	18.3	3.5	58.0
Oshimili South	18.3	4.0	53.5
Bende	18.3	4.7	51.5
Calabar-Municipal	18.3	6.4	48.2
Lagelu	18.4	4.5	52.7
Gwer East	18.5	3.9	55.9
Ibadan South East	18.6	7.3	49.9
Aiyedade	18.7	3.9	57.5
Odo-Otin	18.7	5.7	50.0
Ngor-Okpala	18.8	4.4	54.5
Makurdi	18.8	4.8	54.3
Boluwaduro	18.8	5.7	54.5
Ikpoba-Okha	18.8	3.8	57.3

Akoko North East	18.8	5.0	54.1
Essien Udim	18.9	5.6	48.5
Oturkpo	18.9	4.2	56.8
Aniocha South	19.0	4.6	54.4
Uzo-Uwani	19.0	4.3	55.7
Aniocha North	19.0	4.4	55.3
Ogbomosho North	19.1	5.6	49.6
Ankpa	19.1	4.1	56.8
Tafa	19.2	4.7	54.7
Gwer West	19.3	4.2	56.4
Olamabolo	19.3	4.8	54.0
Tai	19.4	5.5	50.3
Etche	19.4	4.2	58.1
Kuje	19.5	4.3	57.3
Ifo	19.5	5.5	50.5
Ido	19.5	4.1	58.1
Ushongo	19.5	4.2	57.5
Ola-oluwa	19.5	4.4	56.4
Nsit Ibom	19.6	5.4	52.3
Ussa	19.6	4.0	58.9
Nkanu East	19.6	4.9	54.9
Ona-Ara	19.7	5.1	57.7
Ikole	19.8	4.4	57.1
Iwo	19.8	5.6	54.6
Ini	19.8	4.6	56.2
Ezeagu	19.9	5.7	54.3
Buruku	19.9	4.5	58.0
Osisioma Ngwa	19.9	7.4	53.4
Etsako East	19.9	4.3	57.6
Ejigbo	20.1	5.4	54.0
Ibesikpo Asutan	20.1	6.6	51.5
Keffi	20.1	5.0	52.8
Owo	20.1	4.9	56.3
Ika	20.2	5.2	53.3
Ohaozara	20.2	5.0	53.5
Owan East	20.3	4.5	57.9
Emohua	20.3	4.3	58.6
Uhunmwonde	20.3	4.5	58.3
Udu	20.3	5.1	60.1
Badagry	20.3	4.3	57.4
Oyigbo	20.4	5.4	53.6
Ekiti	20.4	5.0	58.0
Pankshin	20.5	4.5	58.5
Dekina	20.5	4.4	59.5
Omumma	20.5	7.1	55.3
Omala	20.5	4.6	58.5
Oshimili North	20.5	4.4	59.1
Ndokwa West	20.5	5.0	56.9
Oredo	20.6	4.3	56.5
Idanre	20.7	4.6	58.9
Oke-Ero	20.8	4.3	59.4
Ijebu North East	20.8	5.0	57.9
Oju	20.9	5.0	57.3
Isoko North	20.9	4.8	57.4
Jema'a	21.0	4.5	60.8
Afikpo South	21.0	5.6	53.7
Ose	21.0	4.6	59.1
Shagamu	21.1	4.6	60.1
Apa	21.1	4.4	61.6
Itu	21.1	4.7	60.1
Isokan	21.2	5.5	57.0
Arochukwu	21.2	6.3	54.6
Owan West	21.3	5.3	57.3
Afikpo North	21.3	6.0	54.9

Lagos Island	21.3	8.3	49.0
Esan South East	21.3	4.8	59.0
Abeokuta South	21.4	10.8	41.8
Ishielu	21.4	5.4	57.5
Ndokwa East	21.4	4.6	61.1
Okene	21.4	4.9	58.2
Kaduna North	21.5	9.3	42.6
Adavi	21.5	4.7	60.9
Ogoja	21.5	5.5	56.8
Etsako Central	21.5	5.1	59.4
Barikin Ladi	21.6	4.8	61.0
Kurmi	21.6	4.5	61.8
Odogbolu	21.6	4.7	61.1
Ogbomosho South	21.6	9.4	41.5
Ado	21.7	4.8	61.0
Oron	21.7	6.7	48.9
Riyom	21.7	5.5	56.7
Konshisha	21.7	4.9	60.0
Bekwara	21.8	4.8	61.3
Aba South	21.8	6.4	56.7
Okrika	21.9	5.1	58.9
Jos East	21.9	5.3	59.5
Onicha	22.0	5.0	61.3
Ijebu Ode	22.0	6.2	52.5
Vandeikya	22.0	5.3	60.7
Lagos Mainland	22.0	15.1	31.5
Ibadan North	22.0	11.5	49.9
Takum	22.1	4.8	61.3
Obafemi-Owode	22.1	5.3	59.9
Ahoada West	22.2	5.4	59.8
Odeda	22.2	5.2	61.2
Orhionmwon	22.2	5.0	61.7
Ukwa East	22.4	5.5	60.4
Anambra East	22.4	5.8	56.8
Abeokuta North	22.4	5.7	59.8
Afijio	22.5	5.2	60.3
Ethiope West	22.5	5.4	60.5
Ohaukwu	22.6	6.2	58.3
Egbeda	22.6	7.9	54.5
Ivo	22.6	5.9	60.0
Ezza North	22.6	5.3	60.0
Sanga	22.7	5.3	60.9
Ijebu North	22.7	5.4	60.8
Yenegoa	22.8	6.4	55.9
Ibarapa East	22.8	5.0	62.9
Ofu	22.8	5.1	62.5
Ethiope East	22.9	5.5	59.7
Orolu	22.9	11.7	47.1
Mkpat Enin	22.9	5.1	60.0
Obi	22.9	4.7	64.7
Aninri	23.0	6.3	58.6
Etinan	23.0	4.5	65.4
Katsina-Ala	23.0	5.1	62.5
Onna	23.0	4.2	65.2
Boki	23.1	4.8	63.6
Eket	23.1	5.3	63.4
Surulere	23.2	5.7	60.9
Ezza South	23.2	7.0	54.8
Etim Ekpo	23.3	5.3	62.2
Okehi	23.3	5.9	60.1
Ewekoro	23.5	5.1	63.4
Tarauni	23.5	7.3	58.4
Mangu	23.6	5.6	63.1
Oyun	23.6	6.3	60.7

Gokana	23.8	8.6	52.3
Egbado South	23.9	5.7	63.4
Ibiono Ibom	24.0	6.5	62.3
Ogbia	24.0	6.1	61.3
Zango-Kataf	24.0	5.5	63.8
Jaba	24.1	5.5	64.0
Abi	24.1	6.2	62.7
Logo	24.1	5.3	64.5
Oyo East	24.2	5.9	61.4
Bassa	24.2	5.4	63.7
Guma	24.2	5.2	64.9
Ipokia	24.2	5.8	70.3
Wamba	24.3	5.5	64.4
Ebonyi	24.4	5.7	63.5
Abua/Odual	24.5	6.3	61.7
Bokkos	24.7	5.9	64.0
Odigbo	24.8	5.9	63.9
Kajola	24.8	6.0	64.5
Nasarawa	24.9	10.4	51.0
Mopa-Muro	24.9	5.4	66.4
Ilorin East	24.9	5.6	64.9
Abakaliki	24.9	5.8	64.3
Sapele	25.0	6.4	64.6
Yala	25.0	5.7	65.3
Gashaka	25.1	5.6	65.0
Yakurr	25.3	6.0	65.4
Igalamela-Odolu	25.3	6.0	64.3
Isoko South	25.4	5.2	67.6
Agatu	25.4	5.9	65.7
Ogu/Bolo	25.4	6.3	63.8
Ibarapa Central	25.5	7.2	63.2
Esit - Eket	25.5	5.8	65.7
Ovia North East	25.5	5.9	65.0
Bassa	25.6	6.1	64.3
Ikom	25.6	5.8	65.7
Kagarko	25.7	5.9	65.8
Ajaokuta	25.7	5.6	66.5
Ukanafun	25.7	5.4	68.5
Offa	25.8	10.7	54.8
Ifako-Ijaye	25.8	7.6	43.9
Okpe	25.9	5.6	67.2
Kabba/Bunu	25.9	6.0	65.7
Ibadan North East	25.9	17.9	36.3
Idah	26.0	9.4	55.1
Degema	26.1	5.1	68.7
Awka North	26.1	6.0	66.7
Nasarawa-Eggon	26.2	6.5	63.9
Karu	26.3	6.4	65.1
Khana	26.4	6.0	65.8
Iseyin	26.4	6.3	66.1
Toto	26.5	6.6	65.2
Uruan	26.6	6.8	64.5
Ibarapa North	26.6	5.3	70.2
Bida	26.8	6.5	65.2
Bonny	26.9	5.1	72.5
Kolokuma/Opokuma	27.0	6.9	63.4
Southern Ijaw	27.1	6.3	67.4
Ekeremor	27.1	6.2	67.6
Akwanga	27.2	6.6	67.6
Obubra	27.2	6.6	66.3
Ughelli North	27.3	7.2	65.0
Ijebu East	27.4	6.4	68.4
Nsit Ubium	27.4	7.2	65.4
Oruk Anam	27.5	6.2	68.7

Yagba East	27.5	7.2	65.6
Kaduna South	27.5	12.0	51.1
Irepodun	27.5	15.0	49.1
Kogi	27.5	6.5	68.2
Toungo	27.6	6.2	68.4
Keana	27.6	6.2	68.8
Etung	27.6	6.1	69.4
Oyo West	27.7	6.8	67.0
Ikwo	27.8	8.0	65.7
Biase	27.8	6.7	68.3
Ibeju/Lekki	27.9	5.5	71.1
Obi	28.1	6.8	68.0
Ukum	28.2	6.8	68.3
Imeko-Afon	28.3	5.8	72.0
Akuku Toru	28.5	6.1	70.8
Nsit Atai	28.5	6.8	71.9
Aba North	28.6	14.7	54.0
Urue-Offong/Oruko	28.7	5.6	71.6
Donga	28.8	6.9	69.1
Yagba West	28.8	6.6	70.2
Ogo Oluwa	28.9	6.5	70.7
Ayamelum	29.1	7.9	67.5
Doma	29.1	7.2	68.9
Ughelli South	29.1	7.2	67.9
Okitipupa	29.1	7.7	67.5
Sagbama	29.2	7.2	69.2
Fagge	29.3	7.5	75.8
Nembe	29.4	7.5	68.4
Izzi	29.5	7.8	68.1
Ikot Abasi	29.6	8.8	66.3
Asa	29.9	7.9	68.5
Iwajowa	30.0	6.9	71.1
Okobo	30.1	7.5	70.5
Kachia	30.1	7.7	70.2
Itesiwaju	30.2	7.1	71.0
Akamkpa	30.2	7.1	71.5
Sardauna	30.3	6.9	71.9
Warri North	30.3	7.2	71.1
Mikang	30.4	7.2	71.3
Kanke	30.5	7.8	71.1
Kokona	30.7	7.7	70.7
Nasarawa	30.8	7.6	70.4
Ifelodun	30.9	8.2	69.4
Odukpani	30.9	7.7	70.7
Kwali	31.1	9.0	68.4
Kano Municipal	31.2	15.2	65.8
Brass	31.2	6.8	73.3
Egbado North	31.3	7.0	73.0
Ovia South West	31.3	7.7	71.4
Patani	31.4	7.9	69.4
Epe	31.4	8.0	70.8
Andoni	31.4	7.2	74.3
Gurara	31.5	7.8	69.9
Ganye	31.5	8.0	70.5
Gwagwalada	31.6	8.8	67.9
Anambra West	31.7	7.7	72.2
Udung Uko	31.7	6.3	76.6
Calabar South	31.9	5.1	76.8
Olorunsogo	31.9	7.8	72.2
Ibaji	32.1	8.3	71.3
Kumbotso	32.1	8.7	68.9
Eastern Obolo	32.1	5.1	80.5
Irele	32.1	8.0	72.5
Lafia	32.5	8.7	71.1

Qua'an Pan	32.6	8.4	72.2
Ibeno	32.7	3.8	84.1
Ibadan North West	32.9	24.7	51.6
Atiba	32.9	8.5	72.3
Akpabuyo	32.9	7.2	73.7
Isu	33.0	17.3	52.1
Ori Ire	33.2	8.8	72.4
Warri South West	33.2	7.7	75.3
Kajuru	33.3	8.5	72.7
Opobo/Nkoro	33.3	13.1	63.2
Hawul	33.4	8.4	73.3
Bomadi	33.7	8.7	72.8
Kauru	33.8	8.6	73.9
Awe	33.9	8.4	74.5
Mbo	34.6	4.9	84.8
Michika	34.8	8.7	74.9
Wukari	34.8	9.1	74.0
Warri South	34.9	8.7	73.8
Bali	35.1	9.1	74.3
Ese-Odo	35.2	8.9	75.2
Ogun waterside	35.2	9.3	74.4
Atigbo	35.2	8.5	76.0
Yola South	35.4	9.3	73.8
Abaji	35.4	9.8	73.2
Jada	35.5	8.6	76.5
Langtang North	35.8	9.1	75.9
Yorro	36.0	9.5	74.6
Lamurde	36.1	9.7	74.8
Burutu	36.1	8.9	76.6
Shendam	36.3	9.5	76.4
Ilaje	36.6	8.2	79.1
Lokoja	36.6	9.9	74.2
Orelope	36.6	8.3	78.4
Bogoro	36.8	9.7	75.8
Lere	36.9	9.5	77.0
Girei	37.2	9.7	75.9
Mubi South	37.3	8.3	78.8
Mubi North	37.4	9.8	76.6
Dass	37.4	9.4	77.3
Jalingo	37.5	9.6	77.2
Zing	37.9	9.4	78.2
Mayo-Belwa	37.9	9.8	77.4
Tafawa-Balewa	38.1	10.6	76.0
Askira/Uba	38.3	10.5	76.9
Chikun	38.4	9.9	77.5
Saki East	38.5	10.8	76.6
Balanga	38.5	10.8	76.4
Fufore	38.9	9.5	79.0
Madagali	38.9	9.7	79.4
Demsa	39.2	10.6	77.8
Ibi	39.4	10.4	78.6
Gombi	39.6	10.3	78.6
Hong	40.0	10.6	78.9
Paikoro	40.1	11.3	78.1
Lapai	40.1	10.4	79.2
Ungogo	40.1	16.9	68.4
Numan	40.1	11.8	77.8
Guyuk	40.3	11.9	77.8
Song	40.9	11.2	79.4
Irepo	41.0	11.0	79.7
Hadejia	41.5	15.9	72.4
Maiduguri	41.8	19.4	69.1
Sokoto South	41.9	16.0	77.1
Sabon-Gari	42.0	13.2	75.6

Biu	42.1	12.0	79.2
Saki West	42.1	11.8	80.1
Langtang South	42.2	11.4	80.6
Shelleng	42.4	13.3	78.5
Moro	42.6	12.0	79.9
Maiha	42.7	10.2	83.2
Bosso	43.0	11.9	80.5
Zaria	43.0	13.8	78.2
Kanam	43.2	12.4	80.7
Shomgom	43.4	13.5	79.2
Toro	43.5	12.7	80.2
Muya	43.6	12.3	81.0
Ardo-Kola	43.8	12.4	80.8
Edati	43.9	12.2	80.9
Bauchi	44.0	12.4	81.4
Kontagora	44.3	12.6	81.1
Katsina	44.8	19.8	75.8
Bakassi	45.1	1.5	97.3
Lau	45.1	13.2	81.5
Kaltungo	45.3	14.3	80.5
Doguwa	45.5	13.4	81.9
Gassol	45.5	13.2	82.1
Pategi	45.9	12.6	83.3
Magama	45.9	13.6	82.2
Zuru	46.2	14.7	80.8
Karim-Lamido	46.5	13.2	83.2
Sakaba	46.5	13.6	82.7
Gombe	46.7	29.0	69.4
Gbako	46.8	13.6	82.7
Mariga	47.0	13.8	83.1
Gwoza	47.1	13.6	83.5
Edu	47.1	13.5	83.3
Agaie	47.2	13.5	83.7
Rijau	47.3	14.1	83.1
Lavun	47.3	14.2	82.8
Kubau	47.4	13.9	83.1
Mokwa	47.5	13.6	83.3
Yauri	47.6	17.3	79.3
Shani	47.8	14.5	82.9
Igabi	47.9	14.6	82.8
Gwale	47.9	25.6	68.7
Chibok	47.9	14.5	83.5
Shiroro	48.2	14.5	83.5
Sokoto North	48.6	18.5	79.5
Markafi	49.0	15.0	83.8
Dala	49.0	20.8	79.2
Kwaya Kusar	49.1	15.6	83.3
Katcha	49.1	14.6	84.3
Wase	49.1	14.4	84.7
Billiri	49.2	16.0	82.5
Wasagu/Danko	49.4	15.0	84.4
Baruten	49.6	14.4	84.9
Madobi	49.6	15.2	84.6
Ningi	49.7	15.2	84.3
Tofa	49.8	15.4	84.1
Rafi	49.9	15.8	84.0
Dawakin Tofa	49.9	17.2	83.2
Alkaleri	50.0	15.0	85.0
Agwara	50.3	15.3	85.0
Ganjuwa	50.3	16.0	84.5
Birnin-Gwari	50.5	14.8	85.6
Gezawa	50.6	15.0	85.6
Funtua	50.7	16.6	83.9
Kura	50.8	16.5	84.3

Garum Mallam	50.8	19.6	81.4
Soba	50.9	16.0	84.9
Mashegu	51.0	15.6	85.4
Fakai	51.1	15.7	85.2
Damboa	51.2	15.8	85.4
Malumfashi	51.4	17.7	83.2
Bichi	51.4	18.2	83.4
Gwarzo	51.4	15.7	85.9
Ngaski	51.5	16.6	85.3
Kafur	51.7	16.0	85.4
Kaiama	51.7	16.5	85.2
Ikara	51.8	16.0	85.8
Akko	51.8	17.3	84.4
Gusau	51.9	16.8	84.9
Wushishi	51.9	16.2	85.7
Maru	52.0	16.2	85.8
Rimin Gado	52.0	14.8	87.1
Kudan	52.1	20.6	82.6
Rogo	52.1	16.4	85.8
Shanga	52.1	15.8	86.3
Tsafe	52.2	16.4	85.8
Bebeji	52.3	16.4	85.7
Shanono	52.5	15.4	87.0
Rimi	52.5	18.7	84.3
Bagudo	52.5	16.6	86.2
Bayo	52.7	17.2	85.0
Batagarawa	52.8	19.2	84.7
Kabo	52.8	18.8	84.0
Minjibir	52.9	18.1	85.1
Giwa	53.0	16.8	86.2
Kibiya	53.0	17.0	86.1
Kiru	53.0	17.6	85.7
Rano	53.3	18.5	85.1
Jere	53.4	17.0	85.6
Karaye	53.4	18.8	85.4
Dawakin Kudu	53.5	18.3	85.8
Bunkure	53.7	17.5	86.4
Faskari	53.7	16.9	87.0
Bagwai	53.9	18.4	86.4
Charanchi	54.0	18.8	85.7
Tsanyawa	54.0	18.7	85.9
Sabuwa	54.1	16.1	87.9
Borgu	54.2	17.4	86.8
Dandume	54.2	18.0	86.4
Bungudu	54.3	17.4	86.8
Tudun Wada	54.3	17.9	87.1
Danja	54.4	18.2	87.0
Warawa	54.5	17.5	87.4
Kankia	54.6	18.1	86.7
Bakori	54.8	18.5	86.7
Yamaltu/Deba	54.9	18.7	86.6
Musawa	55.1	19.7	85.8
Bama	55.1	17.2	87.9
Dutsin-Ma	55.2	17.6	87.5
Kusada	55.2	18.0	87.6
Kirfi	55.3	17.9	87.7
Wudil	55.4	22.2	84.3
Warji	55.4	19.8	86.5
Garko	55.4	19.0	87.4
Kunchi	55.5	19.0	86.9
Gulani	55.8	18.2	87.6
Gummi	55.8	18.7	87.3
Suru	55.9	19.1	87.4
Sumaila	55.9	19.6	86.9

Bindawa	55.9	19.0	87.4
Kwami	56.0	18.5	87.4
Dandi	56.0	18.8	87.1
Roni	56.0	18.1	87.9
Daura	56.0	17.7	87.4
Anka	56.0	18.9	87.4
Makoda	56.1	19.6	87.2
Gwaram	56.2	19.8	86.7
Kazaure	56.2	21.1	85.3
Birnin Magaji	56.3	18.5	87.9
Dan Musa	56.3	19.7	86.7
Mani	56.4	20.9	86.4
Potiskum	56.4	21.3	86.0
Ingawa	56.4	20.6	86.8
Gabasawa	56.5	19.1	87.7
Funakaye	56.5	19.4	87.4
Kankara	56.5	19.5	87.4
Dambatta	56.8	19.4	87.6
Dutse	56.8	19.8	87.1
Ajingi	56.8	18.7	88.3
Kaura Namoda	56.8	20.5	87.0
Jibia	56.9	16.0	90.2
Batsari	57.0	19.1	88.0
Safana	57.0	18.9	88.3
Kiri Kasamma	57.0	19.3	87.7
Kebbe	57.0	18.5	88.5
Gaya	57.1	19.7	87.8
Gujba	57.2	19.9	87.7
Kala/Balge	57.2	16.2	90.3
Konduga	57.3	19.4	88.1
Dikwa	57.4	18.2	89.0
Bukkuyum	57.4	19.9	88.1
Kurfi	57.5	18.9	88.6
Fika	57.5	19.3	88.4
Ringim	57.9	19.7	88.9
Aleiro	58.0	20.1	88.3
Zurmi	58.0	20.0	88.2
Matazu	58.0	22.2	87.5
Birnin Kebbi	58.1	21.6	87.4
Jega	58.1	20.6	88.1
Dukku	58.2	20.7	88.1
Ngala	58.2	17.1	90.4
Kukawa	58.3	19.6	88.9
Sandamu	58.3	22.2	87.8
Talata Mafara	58.4	21.9	87.3
Auyo	58.4	21.5	88.4
Albasu	58.4	20.3	88.7
Koko/Besse	58.4	21.0	88.5
Maiyama	58.5	20.3	88.9
Guri	58.7	18.7	89.6
Nafada	58.7	21.8	87.4
Bunza	58.7	19.7	89.2
Gwiwa	58.8	21.5	88.3
Birni Kudu	58.8	20.9	88.4
Dutsi	58.9	21.5	88.5
Abadam	59.0	18.7	90.0
Kalgo	59.0	22.5	87.3
Damaturu	59.0	20.5	88.6
Wamako	59.0	21.8	87.4
Kaga	59.2	19.8	89.2
Maradun	59.2	21.5	88.4
Kaita	59.2	20.5	88.7
Wurno	59.4	24.4	86.1
Mafa	59.5	21.3	88.8

Kware	59.5	23.6	87.1
Gumel	59.5	20.7	89.1
Shira	59.7	20.6	89.3
Darazo	59.7	20.5	89.3
Kiyawa	59.7	23.3	87.9
Misau	59.8	20.6	89.3
Mai'adua	59.8	22.8	87.5
Monguno	59.9	19.7	90.1
Takai	59.9	22.1	89.4
Miga	59.9	22.3	88.6
Marte	60.0	21.1	89.3
Katagum	60.1	22.7	88.3
Shinkafi	60.1	21.6	89.3
Giade	60.2	23.7	88.1
Yankwashi	60.2	20.5	90.0
Argungu	60.3	23.2	88.1
Damban	60.3	21.8	89.1
Bakura	60.3	22.2	88.7
Fune	60.5	21.9	89.2
Jama'are	60.6	24.1	87.4
Zango	60.7	19.1	90.5
Taura	60.9	21.9	90.0
Mashi	60.9	19.9	90.7
Zaki	61.0	22.4	89.5
Itas/Gadau	61.0	21.7	89.8
Tambuwal	61.1	22.1	89.6
Babura	61.1	23.6	88.6
Sabon Birni	61.2	21.2	89.9
Augie	61.2	22.1	89.6
Garki	61.4	22.8	89.4
Goronyo	61.4	22.0	89.9
Tureta	61.4	21.5	90.1
Buji	61.6	23.2	89.8
Jahun	61.8	22.4	89.7
Arewa-Dandi	61.8	22.1	90.2
Isa	61.8	21.7	90.4
Gwandu	61.9	22.4	90.1
Malam Madori	61.9	23.5	89.6
Gada	62.0	23.7	89.5
Yabo	62.1	23.7	89.5
Bodinga	62.1	23.8	89.1
Nganzai	62.1	22.2	90.3
Kaugama	62.1	23.1	90.0
Bade	62.2	22.3	90.2
Rabah	62.3	21.7	90.6
Illela	62.5	22.1	90.6
Dange-Shuni	62.5	23.7	89.8
Gamawa	62.6	23.7	89.9
Silame	62.6	24.6	89.8
Nangere	62.6	23.9	90.1
Guzamala	62.8	22.3	90.8
Gwadabawa	62.9	25.0	89.4
Shagari	63.0	22.4	90.9
Jakusko	63.0	23.0	90.4
Nguru	63.0	22.6	90.4
Machina	63.1	23.7	90.0
Bursari	63.1	22.7	90.8
Gubio	63.2	23.6	90.5
Tarmua	63.3	23.2	90.7
Sule-Tankarkar	63.3	22.5	90.9
Baure	63.4	21.7	91.4
Binji	63.4	23.6	90.5
Magumeri	63.4	22.7	91.0
Mobbar	63.4	22.5	91.2

Kafin Hausa	63.5	24.5	90.3
Geidam	63.5	23.4	90.8
Gudu	63.6	22.6	91.1
Gagarawa	63.8	22.0	91.7
Tangaza	64.5	23.0	91.7
Karasuwa	65.3	25.4	91.2
Maigatari	65.6	24.1	92.0
Yunusari	65.7	23.4	92.3
Yusufari	66.3	24.4	92.1
Biriniwa	66.4	26.3	91.7
