# Supplementary figures and images for: Mapping Local Variations and the Determinants of Childhood Stunting in Nigeria
Source: Int J Environ Res Public Health. 2023 Feb 13;20(4):3250. doi: 10.3390/ijerph20043250 (PMC9959360; doi:10.3390/ijerph20043250)

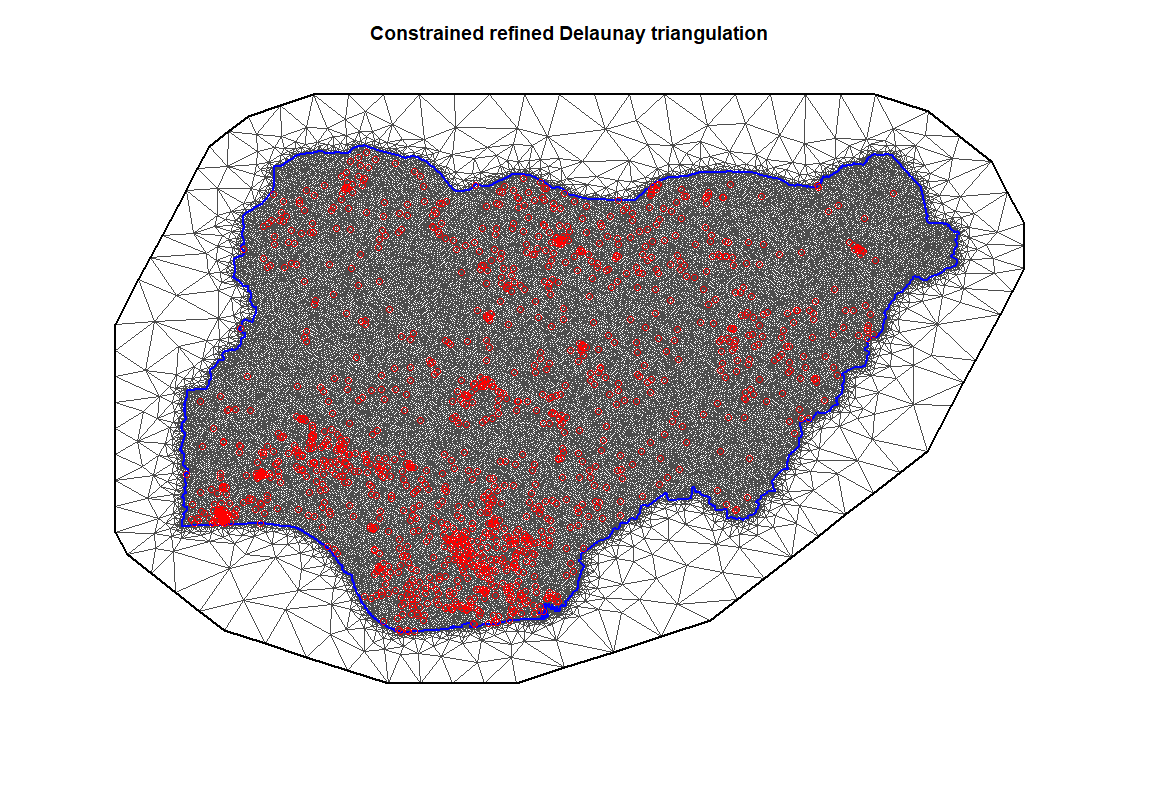

Supplement: Supplementary file 1 [file ijerph-20-03250-s001.zip › Supplementary Figure S1.tiff]

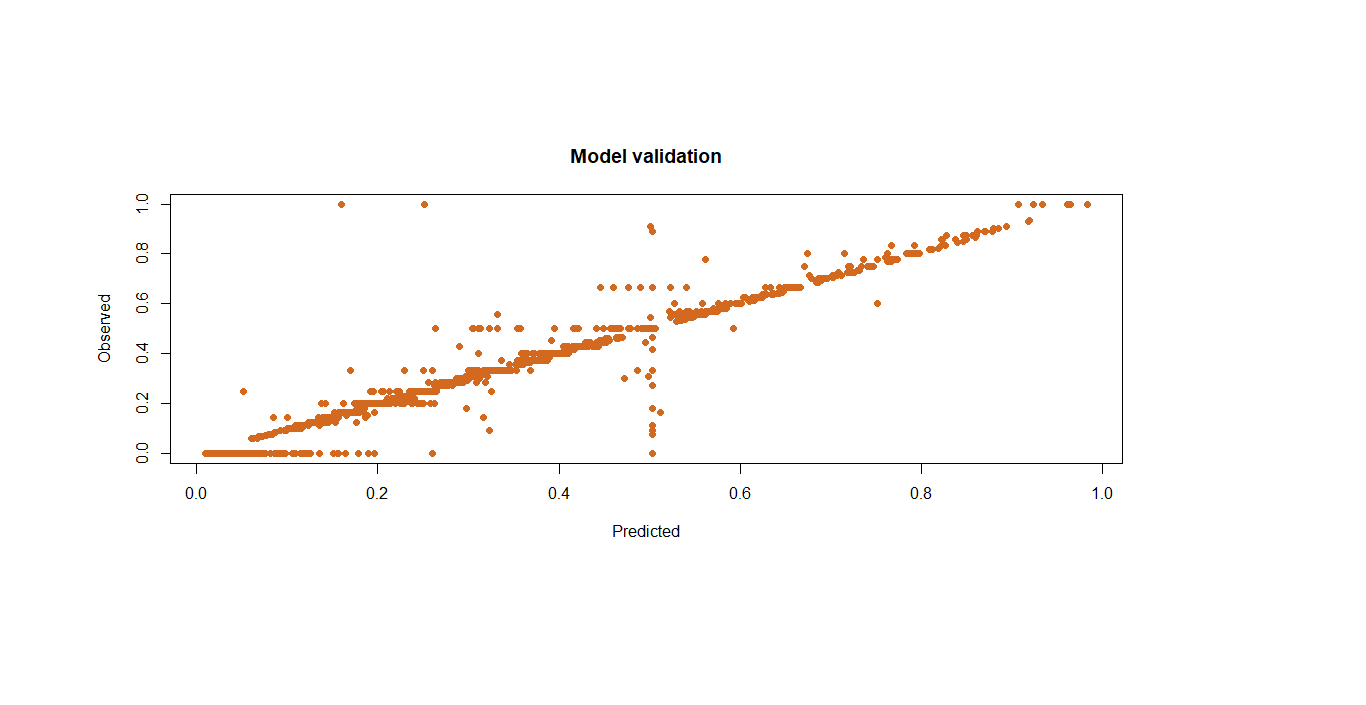

Supplement: Supplementary file 1 [file ijerph-20-03250-s001.zip › Supplementary Figure S2.tiff]
